# Recent Developments in Ferulic Acid- and Caffeic Acid-Based Hybrids with Potential Anticancer Properties

**DOI:** 10.3390/molecules31111875

**Published:** 2026-05-29

**Authors:** Sijongesonke Peter, Linda Lunga Sibali, Vuyolwethu Khwaza, Athandwe M. Paca

**Affiliations:** 1 Department of Chemical and Earth Sciences, Chemistry Discipline, Faculty of Science and Agriculture, University of Fort Hare, Alice 5700, South Africa; vkhwaza@ufh.ac.za; 2 Office of the Dean, Faculty of Science and Agriculture, University of Fort Hare, Alice 5700, South Africa; lsibali@ufh.ac.za

**Keywords:** ferulic acid, anticancer, caffeic acid, hydrocinnamic acids, pharmacophores, hybridization, drug resistance

## Abstract

The shortage of effective chemotherapeutic agents poses a significant challenge to the global public health system. Cancer is among the leading diseases affecting the human population worldwide. Issues such as drug resistance, toxicity, lack of specificity, poor bioavailability and water solubility, and severe side effects reduce the effectiveness of many existing anticancer drugs. As a result, there is growing interest in discovering a new generation of therapeutic agents to overcome these limitations. Phenolic acids, including ferulic and caffeic acids, are cinnamic acid derivatives with numerous biological effects, including anti-inflammatory, antibacterial, antifungal, antioxidant, antiviral, cytotoxic, and antiproliferative effects. In recent years, drug repurposing and hybridization strategies have emerged as attractive approaches in medicinal chemistry because they may reduce both the cost and time associated with conventional drug discovery. As a result, several researchers have combined ferulic acid and caffeic acid scaffolds with different pharmacophores to generate hybrid compounds with enhanced anticancer potential. This review summarizes recent in vitro and in silico studies published between 2022 and 2025 on ferulic and caffeic acid hybrid compounds that exhibit cytotoxic and antiproliferative effects. Furthermore, the review discusses structure–activity relationship trends, synthetic approaches, and structural modifications associated with improved biological activity. Collectively, the findings highlight the significant potential of ferulic acid and caffeic acid scaffolds in the development of multifunctional anticancer agents.

## 1. Introduction

The global burden of both non-communicable and communicable diseases, including cancer, continues to rise, largely due to factors such as drug resistance and limited therapeutic efficacy [1,2,3]. This growing prevalence has placed considerable strain on public health systems, as the numbers of deaths, cases, and hospitalizations contribute to significant socio-economic challenges [4,5,6]. Projections suggest that these numbers will continue to grow until at least 2050 due to the lack of effective drugs, as tumor cells evolve and develop defense mechanisms against current treatments [7,8,9,10]. Drug resistance presents a major challenge for public health, as it turns treatable diseases into incurable ones, thereby reducing the human lifespan [11]. Therefore, there is an urgent need to develop new chemotherapeutics to combat the growing problem of drug resistance.

Natural products have played a critical role in drug discovery due to their structural diversity and wide range of biological activities [12,13,14,15]. It is estimated that eighty to ninety percent of the world population is supported by traditional medicine to treat current diseases, with sixty percent of cancer drugs originating from natural products [16,17]. For instance, positive chemotherapeutic outcomes from natural-origin anticancer drugs, such as paclitaxel, vincristine, camptothecin, etoposide, vinblastine, podophyllotoxin, irinotecan, and topotecan, demonstrate the contribution of natural-origin drugs to the treatment of cancer diseases [16,17]. Therefore, the use of natural-origin compounds to develop novel chemotherapeutic agents is an interesting paradigm for medicinal researchers. Hence, the cytotoxic and antiproliferative effects of ferulic acid (FA) and caffeic acid (CA) are being investigated [18,19].

**Figure 1:**
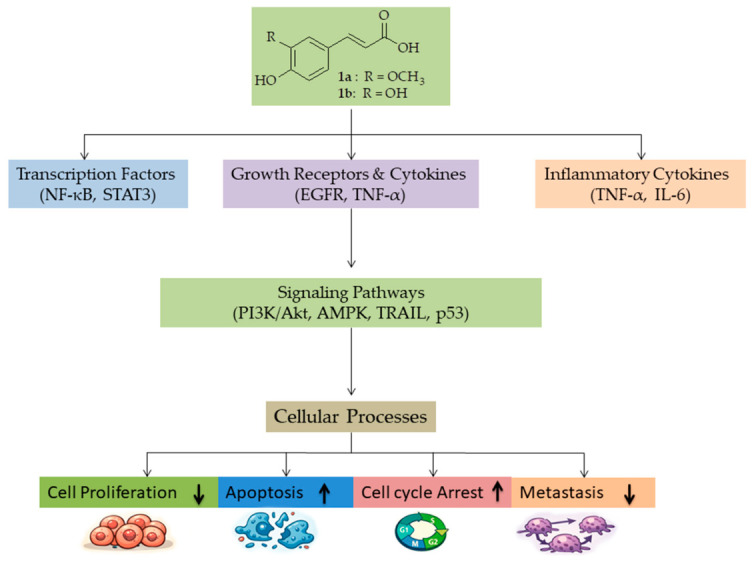
Schematic illustration of the molecular targets and anticancer mechanisms of FA (**1a**) and CA (**1b**). Note: “↑” indicates upregulation or increase; “↓” indicates downregulation or decrease.

FA and CA, depicted in Figure 1 as compounds **1a** and **1b**, respectively, are hydrocinnamic derivatives from the phenolic class of compounds, commonly extracted from angiosperms [20]. Additionally, these two compounds are widely distributed across various dietary sources, such as vegetables, fruits, cereals, grains, and beverages [20,21,22]. They exhibit various biological activities, including anti-inflammatory, antioxidant, cytotoxic, antiproliferative, antimicrobial, and neurological effects, with their hydroxyl groups, aromatic rings, conjugated double bonds, and unsaturated carboxylic functional groups responsible for their biological mechanisms of action [22,23,24,25]. In terms of their potential anticancer properties, FA and CA have been widely reported to exhibit in vitro cytotoxic and antiproliferative effects through their ability to regulate multiple molecular targets. These include cellular transcription factors, growth factor receptors, and inflammatory cytokines, all of which are involved in key processes, such as cell proliferation, cell cycle arrest, metastasis, and apoptosis in cancer cells [26,27,28,29] (Figure 1). Their cytotoxic effects against cancer cell lines have been observed across a range of cancers, including lung, colon [30], prostate [31,32], cervical [33,34], ovarian [35,36], bladder [37], gastric [38,39], renal [40,41], and liver cancers [42,43]. Additionally, FA and CA exert their effects by targeting important signaling pathways, such as the p53 [44,45,46,47], PI3-K/Akt and AMPK pathways [30,48], and key transcription factors, including nuclear factor kappa B (NF-κB) [49,50], Tumor Necrosis Factor-Related Apoptosis-Inducing Ligand (TRAIL) [51,52], and Signal Transducer and Activator of Transcription 3 (STAT3) [30,38,53]. However, their therapeutic potential is limited by factors such as poor bioavailability, low water solubility, limited stability, and potential toxicity [22,54]. Therefore, several medicinal scientists are increasingly adopting the strategy of combination therapy, including the design of hybrid drugs, to overcome the limitations associated with single-entity FA and CA, leading to the discovery of new hybrid compounds with enhanced in vitro antiproliferative activity.

Hybrid drugs are a combination of different pharmacophores linked through various linkers, such as esters, amides, carbamates, imides, and thioesters, using suitable functional groups [55,56,57]. Many medicinal scientists have employed the strategy of drug hybridization to develop new therapeutic drugs as an alternative approach to enhance the biological effects of existing drugs and to treat diseases with complex etiologies, including cancer [55,56,57]. A hybrid drug combination is distinct from polytherapy (PT) and fixed-dose combination (FDC) therapy and overcomes the drawbacks, such as drug–drug interactions, increased toxicity, and side effects, associated with both PT and FDC [54]. Additionally, this approach complements the drug reprofiling strategy, which is the use of known drugs for different therapeutic applications, with both strategies providing better therapeutic outcomes and cost–time efficiency [57]. Therefore, nowadays, combining these strategies can result in better chemotherapeutic agents with fewer limitations and multiple targets [55,56]. Hence, FA and CA are combined with other pharmacophores.

In the drug discovery process, the application of online computational tools (in silico studies) has gained momentum due to their advantages, such as cost- and time-effectiveness [58,59,60,61]. This strategy is widely used as an optimization tool during drug development. It helps predict toxicity, administration routes, drug likeness, pharmacokinetic behavior, and physicochemical properties of newly synthesized compounds. Consequently, in silico approaches complement both drug repurposing and hybridization strategies by reducing development costs and saving time [58,59,60,61]. Hence, the in silico strategy is also an alternative used to reinforce the findings of in vitro evaluations of promising chemotherapeutics. Therefore, chemists are utilizing it in their studies, as it serves as a motivation for further research, including clinical trials of promising therapeutic agents. Notably, this review article focuses on documenting the recent literature on FA and CA hybrid compounds with anticancer activities published between 2022 and 2025. It also focuses on highlighting recent advancements in FA and CA structural modifications, which have resulted in promising chemotherapeutic agents. Furthermore, the synthetic routes used to develop the reported FA and CA hybrids and their influence on the production of novel hybrids are also discussed in this review article.

## 2. Relationship Between Drug Repurposing and the Hybridization Strategy

**Figure 2:**
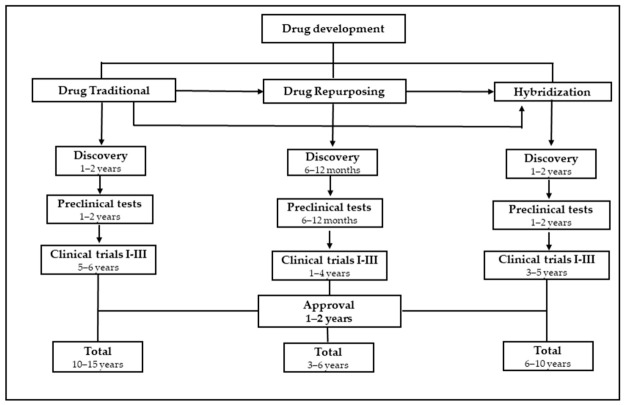
Comparative overview of drug development strategies, highlighting the timelines and cost implications of traditional drug discovery, drug repurposing, and hybridization approaches.

Production of a new drug involves several processes (as depicted in Figure 2), and it takes years, with an estimated investment of $1–2 billion to develop a single drug, with only about one percent of drugs reaching clinical practice and even fewer entering the market [62]. This has led researchers to adopt a drug repurposing strategy to treat current diseases [59,60]. This approach is considered cost- and time-efficient since the pharmacokinetics, pharmacodynamics, and safety data of the drugs are already available [63,64,65,66]. To et al. [67] reported that it takes approximately 14 years to develop a new drug and costs US$2.5 billion, while it takes 3–9 years to approve repurposed therapeutics, with 50–60% savings. This implies a time–cost efficiency of the drug repurposing strategy compared to the traditional development of the drug. However, some repurposed therapeutic agents demonstrate low efficacy, forcing dose increases or the use of multiple drugs in combination, including PT and FDC, leading to increased drug–drug interactions, toxicity, side effects, and reduced patient adherence [63,67]. Moreover, intellectual property issues and marketing constraints are also obstacles that compromise the efficiency of this strategy [68]. Hence, hybridization of these repurposed drugs is among the alternative approaches used to overcome these limitations.

Drug hybridization encourages the use of already known and approved drugs in combination, connecting them via different linkers to improve their biological activity. Therefore, it also improves cost and time efficiency [54,56,69,70]. The hybrid strategy complements the drug repositioning approach by overcoming the limitations associated with drug repurposing, as the new drugs produced via this strategy display reduced drug–drug interactions and toxicity, with the ability to target multiple sites and modes of action [54,56,69,70]. In essence, these two strategies improve time efficiency compared to traditional drug development, as displayed in Figure 2. Hence, combining them is an interesting approach.

## 3. Overview of Ferulic and Caffeic Acids

**Figure 3:**
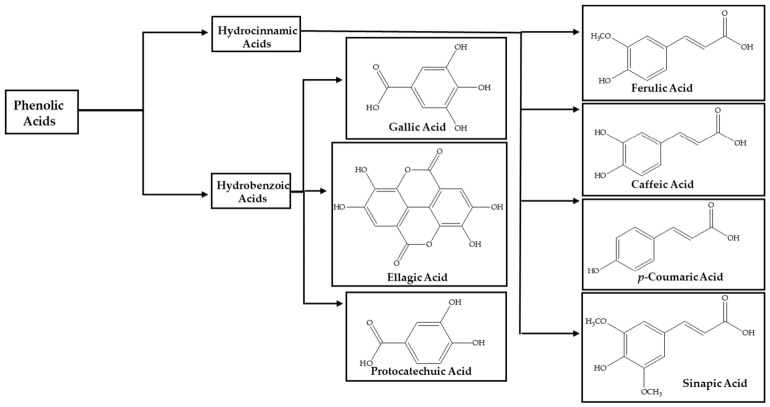
Chemical structures of representative phenolic acids, including FA and CA.

FA and CA are natural bioactive molecules that belong to the main phenolic acid class of compounds, with at least one hydroxyl group and an unsaturated alkyl chain attached to the aromatic ring. They are derivatives of hydrocinnamic acids, as shown in Figure 3 [71,72,73]. These two phenolic compounds are utilized for several industrial applications, such as cosmetics, food, packaging materials, textiles, and pharmaceuticals [74,75]. They possess a wide range of biological activities, including antibacterial, antiproliferative, antioxidant, anti-inflammatory, antifungal, and anti-Alzheimer’s effects, highlighting their significance in the development of novel pharmaceuticals [74,75,76,77]. However, the majority of these biological findings are from in vitro studies, with limited evidence from in vivo applications [73]. Additionally, the biological efficacy of phenolic acids, including FA and CA, is compromised by several factors, such as poor water solubility and bioavailability [22,54]. Therefore, more potent pharmaceuticals containing these phenolic moieties must be explored to enhance their chances of reaching the market. Thus, medicinal scientists are hybridizing them with other pharmacophores to enhance their biological efficacy.

The proposed mechanisms underlying the cytotoxic and antiproliferative effects of these two hydrocinnamic acid compounds (FA and CA) involve numerous processes, including inducing apoptosis, inducing expression of p53, disrupting the tumor’s mitochondrial processes, and inhibiting signaling pathways [19,21,47,78,79,80]. Specifically, FA disrupts cell mitochondrial functions by inhibiting the production of reactive oxygen species (ROS), leading to deoxyribonucleic acid (DNA) damage, resulting in cell death. This compound triggers cell apoptosis and proliferation by enhancing the expression of the p53 tumor protein, resulting in compromised expression of cyclin-dependent kinase (CDK4/6) and cyclin D1, and hinders the expression of antiapoptotic protein Bcl-2 [21]. Moreover, FA disrupts processes such as angiogenesis, tumor migration, and tumor invasion by suppressing PI3K/AKT/mTOR, VEGF mRNA, and fibroblast growth factor (FGF1) signaling pathways [21]. On the other hand, CA displays a similar mechanism to FA. Notably, CA induces cell cycle arrest and apoptosis by improving the expression profile of caspases 1 and 3. Additionally, this compound also promotes the production of CA^2+^, which also affects apoptosis processes. Furthermore, this compound can alter mitochondrial membranes by reducing ∆Ψm by inducing the translocation of PKCδ, resulting in apoptosis [19].

Notably, the biological mechanism of these therapeutic agents is influenced by structural motifs, with carbonyl groups and hydroxyl groups present in FA and CA contributing to their hydrophilicity [81]. This illustrates that they are a key part of the chemical structures of these compounds, and their modification can influence their biological efficacy. These two functional groups are susceptible to being esterified, resulting in novel therapeutic agents. Hence, in the context of developing hybrid compounds, these are two suitable functional groups that must be prioritized in the development of ester- and amide-linked hybrid compounds, as the ester and amide linkers are reported to improve the biological efficacy of the hybrid drugs [82,83]. Thus, several researchers have utilized them as priority modification sites for direct esterification and amidation [81,84].

## 4. Ferulic and Caffeic Acid Hybrid Compounds

**Figure 4:**
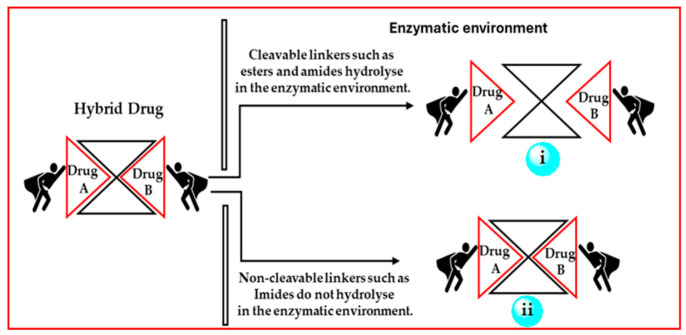
Schematic representation of the design and biological behavior of hybrid compounds, illustrating the combination of two pharmacophores via cleavable (i) and non-cleavable (ii) linkers.

Hybridizing two or more different compounds has been an interesting approach to developing new chemotherapeutics in the 21st century, as this approach displays more benefits than limitations, in contrast to other combination strategies, such as PT and FDC therapy [85]. The positive developments from ferrocifen (a hybrid of ferrocene and tamoxifen) in the treatment of breast cancer have motivated several medicinal researchers to utilize the hybrid approach to overcome issues such as drug resistance and toxicity, drug–drug interactions, poor solubility, and lack of specificity associated with other chemotherapeutic strategies [86]. Hence, medicinal scientists are hybridizing different pharmacophores via both cleavable and non-cleavable linkers to overcome these limitations [79]. Typically, hybrid molecules incorporating two or more pharmacophoric units are generally classified depending on the manner in which these units are covalently linked (Figure 4). For instance, cleavable linkers (i in Figure 4) such as esters and amides can be easily hydrolyzed in the enzymatic environment, promoting the original mechanism of action of drugs and multitarget drugs. This could be beneficial in the treatment of complex cancer disease. On the other hand, non-cleavable linkers (ii in Figure 4), such as imides and other heterocyclic linkers, improve specificity, as the drug binds to one targeting site, resulting in enhanced bioavailability. However, this could result in compromised activity, as the mechanism of action could be modified due to the functional groups responsible for their original mechanism of action being used to hybridize the drugs. Therefore, the type of linker influences the biological activity of hybrid compounds [54,87]. Hence, they must be considered when developing new therapeutic agents.

In the search for new chemotherapeutics, naturally derived compounds make a significant contribution, with numerous health benefits [15]. For instance, positive outcomes from paclitaxel (derived from the Pacific yew tree) led medicinal researchers to use more naturally based compounds to develop new chemotherapeutic agents [88]. However, the progress in the development of new chemotherapeutic agents is disappointing, as the efficacy of cancer therapeutic agents differs from patient to patient [88]. Hence, researchers have hybridized FA and CA with other pharmacophores to develop hybrid compounds with improved in vitro antiproliferative activity and overcome the drawbacks associated with these hydrocinnamic acid compounds.

**Figure 5:**
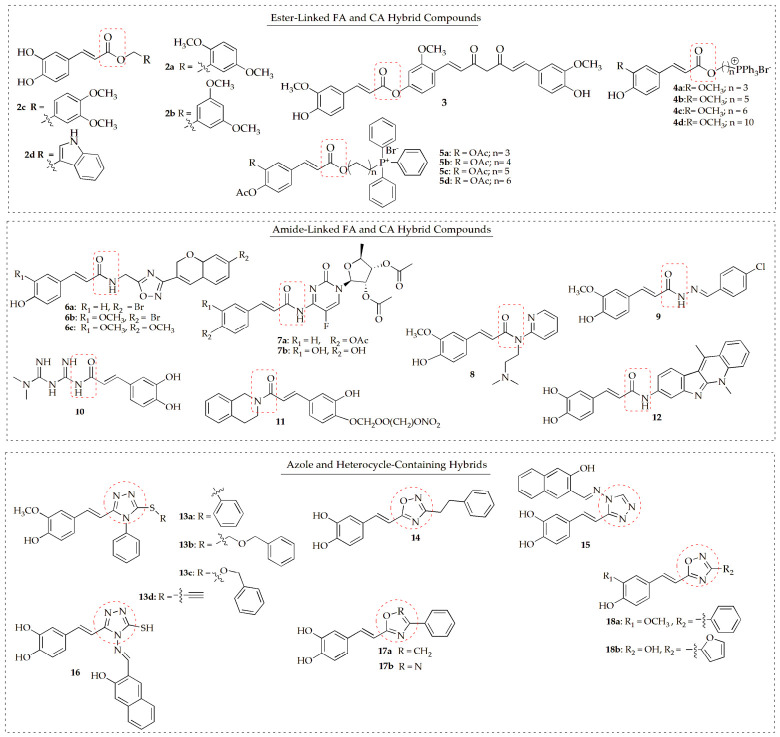
Chemical structures of FA- and CA-based hybrid compounds (**2**–**18**) reported between 2022 and 2025, highlighting the diversity of pharmacophores, linker types (e.g., ester, amide, and azole and heterocyclic linkers), and structural modifications explored for cytotoxic and antiproliferative activity.

Hybridization strategies involving FA and CA derivatives can generally be classified according to the type of linker and pharmacophoric integration used during molecular design (Figure 5). These approaches include cleavable linker systems such as ester- and amide-linked hybrids, non-cleavable heterocyclic hybrids, and bioisosteric modifications. Since linker chemistry and pharmacophore selection strongly influence biological activity, stability, selectivity, and pharmacokinetic behavior, organizing the reported hybrids according to design strategy provides a clear comparative understanding of their advantages and limitations.

### 4.1. Ester-Linked FA and CA Hybrid Compounds

Ester-linked hybrid compounds represent one of the most extensively explored design strategies for the structural modification of FA and CA. In these hybrids, the ester bond serves as a cleavable linker that connects FA or CA scaffolds with additional pharmacophoric units to enhance biological activity and target selectivity. Esterification is particularly advantageous because both FA and CA contain reactive carboxylic acid and hydroxyl functional groups that can readily undergo coupling reactions under mild synthetic conditions. Moreover, ester linkers can facilitate intracellular hydrolysis, potentially releasing active pharmacophoric fragments within the cellular environment and thereby promoting multitarget biological effects [89,90].

Despite these advantages, ester-linked hybrids are often associated with metabolic instability due to enzymatic hydrolysis by esterases, which may compromise their pharmacokinetic behavior and reduce systemic stability [91]. Consequently, several studies have attempted to balance the improved in vitro antiproliferative activity of ester-linked systems with strategies aimed at enhancing their metabolic stability.

Among the reported ester-linked hybrids, Consoli et al. [92] synthesized a series of caffeic acid phenethyl ester (CAPE)-inspired hybrids to improve the biological properties of CAPE while maintaining its mechanism of action. CAPE is recognized for its ability to inhibit cancer cell migration and induce ferroptosis through modulation of heme oxygenase activity [93]. The synthesized compounds, particularly hybrids **2a**–**d** (Figure 5), demonstrated notable antiproliferative effects against MDA-MB-231 breast cancer cells in vitro. Compound **2b** exhibited the strongest cytotoxic effect among the tested derivatives and was reported to induce ferroptosis-associated cellular responses, including glutathione depletion, increased lipid hydroperoxide accumulation, and mitochondrial dysfunction through elevated reactive oxygen species production. Compound **2b** was also evaluated in silico using pkCSM and SwissADME, which showed its ability to block HERG K^+^, illustrating its ability to trigger cardiotoxicity. Interestingly, it displayed good drug-likeness properties, with no Egan’s, Ghose’s, Lipinski’s, Veber’s, or Muege’s rule violations. The compound showed no BBB permeability, illustrating that it could not be toxic to the CNS. Furthermore, this compound was predicted not to be a P-glycoprotein (Pg)-substrate. However, this compound displayed characteristics of Pan-Assay Interference Compounds (PAINS) due to the presence of two hydroxyl groups. Interestingly, the presence of dimethoxy substituents on the aromatic side chain enhanced the observed biological activity, emphasizing the importance of electron-donating groups in improving interactions with biological targets.

Similarly, Cheng et al. [94] synthesized an ester-linked FA–curcumin hybrid (**3**, Figure 5) using Steglich esterification between the carboxylic acid group of FA and the hydroxyl group of curcumin. The hybrid demonstrated improved in vitro antiproliferative activity against several cancer cell lines, including A549, HepG2, HeLa, and MCF-7, compared to its parent compounds. Notably, compound **3** displayed stronger growth-inhibitory effects against A549 lung cancer cells than cisplatin (Table 1), suggesting that hybridization enhanced the biological performance of both pharmacophores. Mechanistically, the hybrid was reported to suppress signaling pathways associated with cell proliferation and migration, including PI3K, VEGFR2, EGFR, and mTOR pathways, while also promoting apoptosis and cell cycle arrest. These findings illustrate how ester-linked hybridization may improve multitarget biological activity through synergistic pharmacophore integration.

Mitochondria-targeting ester-linked hybrids have also attracted significant attention. FA targets the mitochondria of the cell and disrupts its processes, leading to cell death, and triphenylphosphonium salts are reported to improve the delivery of drugs to the mitochondria of cancer cells. Hence, Wu et al. [95] synthesized triphenylphosphonium-containing FA hybrids (**4a**–**d**, Figure 5) to enhance mitochondrial delivery and improve intracellular accumulation. The introduction of triphenylphosphonium salts significantly enhanced the in vitro cytotoxic effects of the hybrids compared to FA alone. Collectively, phosphonium-containing hybrids suggest that mitochondrial targeting significantly enhances antiproliferative activity, likely through improved intracellular accumulation and ROS-mediated apoptosis. Mechanistic studies suggested that the compound disrupted mitochondrial function, promoted apoptosis through Bcl-2/Bax/Caspase-3 signaling, increased reactive oxygen species generation, and inhibited cell migration. Importantly, the study also demonstrated that longer alkyl chain lengths between the FA and phosphonium moieties improved lipophilicity and biological activity.

Comparable findings were reported by Lukáč et al. [96], who synthesized phosphonium-containing CA ester hybrids (**5a**–**d**, Figure 5). These compounds exhibited stronger antiproliferative effects than CA across several cancer cell lines, including HeLa, HCT116, and MDA-MB-231 cells. The improved activity was attributed to enhanced mitochondrial targeting and increased membrane permeability associated with the phosphonium moiety. Furthermore, elongation of the alkyl linker chain positively influenced biological activity, reinforcing the importance of lipophilicity and intracellular delivery in the design of ester-linked hybrids.

Overall, ester-linked FA and CA hybrid compounds have demonstrated considerable potential as multifunctional anticancer agents due to their ability to integrate complementary pharmacophores within a cleavable molecular framework. The reviewed studies consistently showed that esterification improved cellular uptake, mitochondrial targeting, and multitarget biological activity, often resulting in enhanced antiproliferative effects compared to the parent compounds. Structural features such as methoxy substituents, phosphonium salts, and extended alkyl chains frequently contributed to improved activity through enhanced lipophilicity and intracellular accumulation. However, despite their promising in vitro and, in some cases, in vivo performance, ester-linked hybrids remain limited by their susceptibility to enzymatic hydrolysis and metabolic instability. These findings suggest that although ester linkers are advantageous for promoting synergistic pharmacological effects and intracellular drug release, additional optimization strategies are required to improve their pharmacokinetic stability and therapeutic applicability.

### 4.2. Amide-Linked FA and CA Hybrid Compounds

Amide-linked hybrid compounds constitute another widely investigated strategy in the structural modification of FA and CA. Compared to ester linkers, amide bonds generally exhibit superior chemical and metabolic stability due to their reduced susceptibility to enzymatic hydrolysis. This enhanced stability may improve the pharmacokinetic behavior, bioavailability, and systemic persistence of hybrid compounds. Furthermore, the amide functional group can actively participate in hydrogen bonding interactions with biological targets, thereby enhancing binding affinity and selectivity toward specific biomolecular receptors or enzymes.

However, despite their improved stability, amide-linked hybrids may sometimes display lower biological potency compared to ester-linked analogs due to reduced intracellular cleavage and diminished release of active pharmacophoric fragments. Consequently, the biological performance of amide-linked FA and CA hybrids strongly depends on the nature of the incorporated pharmacophore, substitution pattern, and overall molecular architecture.

Jadhaw et al. [97] synthesized a series of amide-linked chromene–oxadiazole phenolic acid hybrids incorporating FA scaffolds and evaluated their in vitro cytotoxicity against several cancer cell lines, including HEK-293, HEPG2, B16, SKBR3, and DU145. Among the synthesized compounds, hybrids **6a**–**c** (Figure 5) demonstrated moderate to strong antiproliferative effects, with compound **6b** exhibiting the most favorable activity profile, as indicated by its lower IC_50_ values (Table 2). The improved biological activity was associated with the presence of methoxy and bromo substituents, which likely enhanced hydrophobic interactions and electronic effects involved in target binding [98]. In silico drug-likeness evaluations further demonstrated favorable oral bioavailability and acceptable physicochemical properties, supporting the potential of these amide-linked hybrids as promising lead compounds for further investigation.

Cybulski et al. [99] developed amide-linked hydrocinnamic acid hybrids containing CA moieties and evaluated them against pancreatic cancer cell lines (AsPC-1 and BxPC-3). Among the synthesized hybrids, two caffeic-based hybrids (**7a**–**b**, Figure 5) exhibited no significant effects against AsPC-1. In contrast, they yielded promising outcomes on BxPC3 cancer cell lines, with IC_50_ values of 31.59 µM and 23.16 µM, superior to capecitabine (IC_50_ > 200 µM) and inferior to 5-fluorouracil (IC_50_ = 12.74 µM), in vitro (Table 3). Additionally, they showed no significant toxicity towards normal cells (NHDFs), with IC_50_ values of 121.30 µM and 163. 40 µM, respectively. Furthermore, hybrids **7a** and **7b** were selective towards cancer cells, with selectivity indices of 1.35 and 3.83 towards AsPC-1 and 3.83 and 7.06 towards BxPC3, respectively. This reveals that these compounds are more selective towards BxPC3 than AsPC-1.

Molecular docking studies suggested that the amide linker contributed significantly to hydrogen bonding interactions with amino acid residues such as Ser228, potentially enhancing target affinity and selectivity. Notably, the presence of free hydroxyl groups improved the observed biological activity, emphasizing the importance of preserving key functional groups during hybrid design. Although these compounds displayed promising selectivity profiles, in silico predictions raised concerns regarding potential hERG-mediated cardiotoxicity, highlighting the necessity for further toxicological evaluation.

Another notable example of amide-linked FA hybrids was reported by Yang et al. [100], who synthesized chloropyramine–cinnamic acid hybrids targeting focal adhesion kinase pathways in triple-negative breast cancer cell lines. Hybrid **8** (Figure 5) demonstrated strong antiproliferative effects against MDA-MB-231, MDA-MB-157, and MDA-MB-453 cells while displaying comparatively lower toxicity toward normal breast epithelial cells (MCF-10A) (Table 4). The enhanced activity was attributed to the presence of both hydroxyl and methoxy substituents on the cinnamoyl moiety, illustrating the importance of maintaining electron-donating functional groups within the molecular framework. The findings also demonstrated that hybridization with chloropyramine significantly improved the biological profile of the resulting compound compared to the parent drug.

Fonseca et al. [101] synthesized an amide-linked FA-containing hybrid compound (**9**, Figure 5) and evaluated its in vitro antiproliferative effects against A549 and H1299 lung cancer cell lines. This hybrid demonstrated promising anticancer activity, with IC_50_ values of 23.64 µM and 32.15 µM, which are comparable to those of cisplatin (28.13 µM and 32.15 µM), against A549 and H1299 cancer cells, respectively (Table 5). A significant difference was observed in normal cells, as compound **9** displayed superior anticancer activity compared to cisplatin, showing less toxicity towards the normal cells. This suggests that it could replace cisplatin in the treatment of lung cancer. The structure–activity relationship (SAR) displayed a similar trend to that reported by Silva et al. [102]. The position of the chlorine substituent on the aromatic ring significantly influenced biological activity, emphasizing the importance of substitution pattern optimization in amide-linked hybrid systems. Additionally, this hybrid was reported to mechanistically promote a senescence-like state via a reduction in c-Myc and cyclin B1 and upregulation of the p21 and cyclin D1 pathways, leading to the disruption of mitotic progression.

Banerjee and co-workers [103] also reported amide-linked CA–metformin hybrids, designed to combine the metabolic effects of metformin with the cytotoxic properties of phenolic acids. The hybrids were evaluated against two cancer cells, specifically breast (MDA-MB-468) and lung (A549) cancer cell lines, using 5-fluorouracil as a reference drug in vitro. Among the synthesized hybrids, caffeic-based hybrid **10** (Figure 5) demonstrated strong in vitro antiproliferative effects compared to its counterparts, with IC_50_ values of 4.42 ± 2.15µM and 5.47 ± 2.72µM, outperforming the reference drug against A549 cells and showing slightly lower potency than the reference drug against MDA-MB-468 cells (Table 6). Notably, metformin is well-known as an antidiabetic compound with the ability to boost the immune system, but it is not a robust anticancer agent. The study illustrated how drug repurposing and hybridization strategies may synergistically improve biological activity by integrating multiple pharmacological mechanisms within a single molecular framework.

Additionally, Chen et al. [104] synthesized FA–tetrahydroisoquinoline hybrids linked through amide and ester functionalities and evaluated them against normal cells (HUVECs) and several cancer cells (HepG2, HCT-116, A549, and HL-60). These hybrids displayed activity comparable to that of the reference drug (gefitinib) in HUVECs. Among the ferulic-based compounds, compound **11** (Figure 5) exerted notable antiproliferative effects against A549 with a low IC_50_ value, which was comparable to that of gefitinib (Table 7). It is worth noting that, against HepG2, this hybrid is able to disrupt Ras/Raf/MEK and PI3K/Akt/mTOR and downregulate both p-ERK1/2 and p-AKT, resulting in cell death. These findings further support the ability of amide-containing FA hybrids to interfere with multiple oncogenic signaling cascades.

Cybulski et al. [105] synthesized biologically active 5,11-dimethyl-5H-indolo [2,3-b] quinoline–hydrocinnamic hybrid compounds. Two synthetic routes were employed to develop these hybrids using two different protecting groups: acetyl and allyl protective groups. Among the synthesized hybrids, caffeic-based hybrid **12** (Figure 5) exhibited poor anticancer activity against AsPC-1, BxPC-3, MCF-7, and HeLa compared to the parent drug, 5,11-dimethyl-5H-indolo[2,3-b] quinolone (DiMIQ) (Table 8). Moreover, the molecular docking results obtained in Autodock 4.2 showed that the hybrid displayed good docking binding energies compared to the reference drugs cryptolepine, levofloxacin, and etoposide; its mechanism lies in binding to DNA/topoisomerase II, inhibiting cell DNA replication and leading to cancer cell death. In silico ADMET studies using QikProp 4.6 software (Schrodinger Inc., New York, NY, USA) identified hybrid **12** as a promising drug candidate, with a molecular weight of 500 Da, indicating good oral bioavailability. Additionally, the predictions indicated that this hybrid does not violate Lipinski’s rule of five, indicating good drug likeness. However, minor solubility issues were predicted, demonstrating that the compound does not meet Jorgensen’s rule of three. Furthermore, the hybrid was predicted to show cardiac toxicity, as it was predicted to be a HERG K^+^ blocker. In contrast, the toxicity prediction revealed high LD_50_ values, demonstrating that the drug does not pose any toxicity threats, except for moderate mutagenicity and high immunotoxicity.

In summary, amide-linked FA and CA hybrid compounds exhibited several favorable characteristics, including enhanced chemical stability, improved selectivity toward cancer cells, and stronger target-binding interactions mediated through hydrogen bonding. Compared with ester-linked systems, amide-containing hybrids generally displayed superior metabolic stability and more consistent drug-likeness profiles, supporting their suitability for further pharmaceutical development. The biological activity of these hybrids was strongly influenced by the nature of the incorporated pharmacophores, substitution patterns, and preservation of key hydroxyl functionalities. Several compounds demonstrated promising antiproliferative activity comparable to that of standard chemotherapeutic agents while showing lower toxicity toward normal cells. Nevertheless, the anticancer potency of amide-linked hybrids varied considerably across studies, and many findings remain limited to in vitro evaluations and computational predictions. Therefore, additional in vivo validation, mechanistic investigations, and pharmacokinetic studies are still necessary to fully establish the therapeutic potential of amide-linked FA and CA hybrids.

### 4.3. Azole- and Heterocycle-Containing Hybrids

The incorporation of azole and other heterocyclic moieties into FA and CA hybrid systems represents an important strategy aimed at improving molecular stability, biological selectivity, target affinity, and pharmacokinetic behavior. Heterocyclic scaffolds such as oxadiazole-, triazole-, pyrazole-, and imidazole-containing systems are frequently used in medicinal chemistry because they can serve as bioisosteric replacements for metabolically unstable ester functionalities while simultaneously enhancing hydrogen bonding capacity, lipophilicity, and electronic interactions with biological targets [106,107,108,109].

Compared to ester-linked hybrids, azole-containing systems are generally more resistant to enzymatic hydrolysis and therefore may exhibit improved metabolic stability and prolonged biological activity. In addition, heterocyclic rings can influence molecular planarity and electronic distribution, thereby modulating interactions with enzymes, receptors, and nucleic acids involved in cancer cell proliferation [110]. Nevertheless, the increased structural rigidity and synthetic complexity of heterocyclic hybrids may occasionally reduce cellular permeability or increase production costs, representing important considerations during drug development.

One of the earliest examples of this approach was reported by Jadhaw et al. [97], who synthesized chromene–oxadiazole hybrids containing FA-derived pharmacophores. The oxadiazole ring served as a stable heterocyclic linker that enhanced the overall rigidity and drug likeness of the compounds. Several hybrids demonstrated moderate to strong antiproliferative effects against cancer cell lines, including HEPG2, DU145, and SKBR3 (Table 2). Among the synthesized derivatives, compound **6b** exhibited the most favorable activity profile, which was attributed to the presence of electron-donating methoxy groups and hydrophobic substituents that improved target interactions. Importantly, the oxadiazole moiety also contributed to favorable predicted pharmacokinetic properties, including acceptable oral bioavailability and membrane permeability.

Malik et al. [111] hybridized two anticancer active pharmacophores (FA and triazole-heterocyclic compounds), resulting in four hybrid compounds (**13a**–**d**, Figure 5), and evaluated them on breast (MCF-7) and lung (A549) cancer cells using tamoxifen and erlotinib as reference drugs in vitro and in silico. In vitro, hybrids **13a**–**c** were the most promising anticancer active hybrids compared to the reference drugs against both cancer cells, with IC_50_ values listed in Table 9. Notably, hybrid **13b** was the most anticancer-effective hybrid among those synthesized, with an IC_50_ value of 200.31 µM against MCF-7 cancer cells.

In silico, these hybrids showed no violation of Lipinski’s rule, a bioavailability score of 0.55, and Topological Polar Surface Area (TPSA) values of less than 140 Å. These findings indicate that hybrids **13a**–**d** are promising pharmaceutical candidates, as they exhibit an acceptable TPSA value and a bioavailability score of more than 0.55, the threshold for a good drug. Moreover, the hybrids pose no toxicity threats to the central nervous system (CNS), as they exhibited no potential to cross the blood–brain barrier (BBB) [111]. Lastly, they exhibited good protein binding energies, characterized by positive interactions when docked with the Epidermal Growth Factor Receptor (EGFR) and Estrogen Receptor alpha (ER-α) [111]. Therefore, compound **13b** could be a potential treatment for breast cancer, as it is the most promising compound compared to its counterparts in vitro and in silico. Hence, more studies, such as in silico toxicology and in vivo studies, are recommended, as these are important steps in testing drug safety during drug discovery [111,112].

Similarly, Robichaud and co-workers [113] combined CA and N′-hydroxy-3-phenylpropanamidine via the oxadiazole linker, resulting in hybrid **14** (Figure 5). The anticancer activity was evaluated in vitro across different cancer cell lines. Additionally, in silico ADME studies of this hybrid were conducted to assess its physicochemical properties, drug likeness, and lipophilicity using SwissADME web tools. The biological limitation of CAPE in the enzymic environment is that it degrades to CA due to the poor metabolic stability of esters [113,114]—hence the introduction of the oxadiazole linker, as oxadiazoles are used as bioisosteres of esters. The incorporation of the oxadiazole scaffold significantly improved biological activity relative to the parent compounds. In silico analysis showed that compound **14** and CAPE had similar results, with no significant difference in lipophilicity (CLogPo/w). This compound exhibited one less rotatable bond, one more hydrogen bond acceptor, and the same number of hydrogen donors as CAPE. This indicates a good drug likeness and supplements the in vitro findings. The enhanced activity was associated with the presence of electron-withdrawing substituents on the aromatic ring, suggesting that both electronic and steric factors influenced the biological behavior of the hybrids.

Triazole-containing hybrids have also received considerable attention due to the stability and versatility of the 1,2,3-triazole ring system. The triazole moiety can act as a rigid bioisosteric linker while simultaneously improving hydrogen bonding interactions and metabolic resistance [115]. In one study, Sehrawat et al. [116] synthesized triazole-linked CA hybrids using click chemistry approaches and evaluated their biological effects against human cervical and breast cancer cell lines. The 1,2,4-triazole rings were introduced on the carboxylic acid group of CA scaffolds. Among the synthesized hybrids, compound **15** (Figure 5) was the most effective, with an IC_50_ value of 8.53 µM (Table 10). However, it was less effective when compared to the reference drug doxorubicin (IC_50_ = 3.62 µM) in vitro. Notably, molecular docking studies using Maestro Glide software 13.1 indicated that the hydroxyl group of caffeic acids plays an important role in forming the hydrogen bonds and binds to key amino acids, including Ser59, Glh30, Phe34, and Phe31, of the targeted protein. Hence, their destruction is not recommended when developing new CA hybrid compounds. Additionally, in silico ADMET studies, conducted via the ADMETlab 2.0 online tool, of hybrid **15** showed that this compound does not violate Lipinski’s rule, it is a non-blocker of HERG K^+^, and it cannot cross the BBB, with no heart or CNS toxicity threats, and demonstrates an acceptable drug likeness. Molecular docking studies suggested that the triazole ring facilitates stable interactions with key amino acid residues within target proteins involved in cancer cell survival and proliferation.

Consistent outcomes were observed for another series of DHFR inhibitors containing a CA moiety, synthesized by Sehrawat et al. [117]. In vitro, hybrid compound **16** (Figure 5) was the most active hybrid in the in vitro cytotoxicity assays compared to its counterparts and methotrexate (reference drug) against MCF-7 cancer cells, with an IC_50_ value of 5.37 µM, compared to 18.96 µM for methotrexate (Table 10). Molecular docking studies using Schrodinger’s Maestro Glide software showed that hybrid **16** binds in a similar way to hybrid **15** with the amino acid residues. Additionally, the in silico ADMET results obtained from the ADMETlab 2.0 online tool also corroborated those reported by Sehrawat et al. [116]. However, hybrid **16** raises cardiac failure concerns, as it was predicted to be a possible blocker of HERG K^+^.

Sucu et al. [118] synthesized a series of hybrid compounds. The authors converted the developed amide-linked hybrid compounds and assessed cell viability against two glioblastoma cell lines (LN229 and T98G) in vitro. The amide-linked hybrid yields poor results, leading to the use of oxazole, oxadiazole, and triazole as bioisosteres to develop new CA derivatives. Thus, two hybrid compounds (**17a**–**b**, Figure 5) displayed improved cell viability percentages, with compound **17b** identified as the most potent hybrid in terms of antiproliferative activity, with IC_50_ values of 14.23 µM and 46.42 µM compared to 112.5 µM and 97.92 µM for CAPE against LN229 and T98G cancer cells, respectively (Table 11). Furthermore, the cleavage of Poly (ADP-ribose) polymerase family proteins is reported to trigger apoptosis. Therefore, hybrid **17b** is reported to increase this process by 4-fold compared to CAPE at 50 µM against the LN229 cell lines.

Moreover, a molecular docking evaluation via the Glide docking program demonstrated that it binds to the DNA binding site of the p50 subunit of NF-κB via hydrogen bonding using the hydroxyl groups of the catechol moiety, illustrating the importance of this moiety in these hybrid compounds. Furthermore, in silico ADME studies using the Swiss server showed that this hybrid had acceptable water solubility, with LogS of −4.12, displayed no toxicity in normal tissue, and had no characteristics that promote cancer growth. Additionally, it was predicted to be a good drug candidate with acceptable lipophilicity and TPSA. The compound was predicted not to be BBB-permeant, demonstrating that it could not be toxic to the CNS.

Another interesting study by Sucu et al. [119] led to the development of a new generation of CA- and FA-based hybrid compounds utilizing oxadiazoles as bioisosteres. These compounds helped to identify the effect of using the catechol moiety as a modification site and the influence of using 1,2,4-oxadiazole and 1,3,4-oxadiazole as bioisosteres on the anticancer activity of hydrocinnamic acid hybrids. Hence, the synthesized compounds were evaluated on different cancer cells. Notably, hybrids **18a**–**b** (Figure 5) exhibited better cytotoxic activity against glioblastoma cancer cells compared to their counterparts, CA and CAPE, with IC_50_ values listed in Table 12. Hybrid **18b** displayed superior cytotoxic activity compared to the reference compounds CA and CAPE, especially against ovarian cancer cells. The findings demonstrate that hybrid **18b** has potential for further anticancer investigation, and the synthesized hybrid compounds are selective towards cancer cell lines. Hence, more cancer cells could be employed to further test the anticancer activity of these compounds.

Collectively, azole- and heterocycle-containing FA and CA hybrids have emerged as promising alternatives to conventional ester-linked systems due to their improved metabolic stability, enhanced molecular rigidity, and favorable pharmacokinetic characteristics. The incorporation of heterocyclic scaffolds such as triazoles and oxadiazoles frequently enhances hydrogen bonding interactions, target affinity, and resistance to enzymatic degradation, thereby contributing to improved antiproliferative activity and drug-likeness profiles. Several studies also highlighted the importance of preserving catechol and hydroxyl moieties to maintain biological activity and effective molecular interactions with cancer-related targets. Despite these advantages, the increased structural complexity of heterocyclic hybrids may complicate synthetic accessibility and optimization, while the majority of reported findings remain restricted to in vitro and in silico evaluations. Consequently, more comprehensive in vivo studies, toxicological investigations, and mechanistic validations are required to determine the translational potential of these heterocyclic FA and CA hybrid systems as clinically relevant anticancer agents.

The hybridization of FA and CA with diverse pharmacophores represents a promising strategy to address key limitations of conventional chemotherapy, including drug resistance, poor selectivity, and suboptimal pharmacokinetics. While many of the reported hybrids demonstrate enhanced in vitro anticancer activity and improved drug-likeness profiles, the overall body of evidence remains largely preliminary and fragmented.

A major strength of these hybrids lies in their multitarget mechanisms, involving apoptosis induction, modulation of signaling pathways (e.g., PI3K/Akt, EGFR, mTOR), and mitochondrial disruption. However, these mechanisms are often superficially explored, with limited mechanistic validation beyond molecular docking or indirect biochemical assays. Additionally, although linker chemistry (cleavable vs. non-cleavable) is highlighted as a key determinant of activity, there is no systematic comparative analysis to clearly establish structure–activity relationships across studies.

Importantly, many compounds show moderate potency when compared to standard chemotherapeutics, and in several cases, their activity remains inferior or only comparable to that of existing drugs. While some hybrids exhibit selectivity toward cancer cells, this is not consistently evaluated across studies, and toxicity profiles remain inadequately characterized, with recurring concerns, such as predicted hERG-mediated cardiotoxicity.

Furthermore, the heavy reliance on in silico ADME predictions and limited in vitro assays raises concerns about the translational relevance of these findings. Only a few studies extend to in vivo validation, and even fewer address critical parameters such as pharmacokinetics, bioavailability, and long-term safety.

Overall, while FA and CA hybrids demonstrate potential as multifunctional hybrid compounds with potential anticancer applications, the current research is constrained by: lack of comprehensive SAR integration, insufficient mechanistic depth, and limited in vivo and toxicological validation.

## 5. SAR Discussion

### 5.1. Impact of Functional Groups on the Anticancer Activity of FA and CA Hybrids

The functional groups present in the reported hybrid compounds were instrumental in determining their antiproliferative activity. Hybrids synthesized by Jadhaw et al. [97] demonstrated that the presence of methoxy or bromo groups on either side of the chromene and FA moieties resulted in enhanced antiproliferative activity of the compounds. This trend was also observed on hybrids synthesized by Nikolova-Mladenova et al. [98]. Thus, hybrid **7b**, with both bromo and methoxy groups, exhibited enhanced cytotoxic effects compared to those with only one functional group. A similar trend was observed for the hybrids synthesized by Consoli et al. [92], where a dimethoxy group on the side chain of the aromatic rings of compounds **2a**–**b** resulted in improved antiproliferative activity of the hybrids. Additionally, the methoxy group influences the potential anticancer activity of the compounds. For instance, observations of hybrids synthesized by Sucu et al. [119] demonstrated that replacement of the methoxy group with a trifluoromethoxy group compromised the antiproliferative activity of the hybrids in vitro. Additionally, both the hydroxyl and methoxy groups play a significant role in the antiproliferative activity of cinnamic acid derivatives, as molecular docking evaluation of hybrids conducted by Sucu et al. [118] showed that the hybrids bind to the DNA binding site of the p50 subunit of NF-κB via hydrogen bonding using the hydroxyl groups of the catechol moiety, illustrating the importance of the hydroxyl group for the interaction of these hybrids with amino acid residues. Additionally, the results from the hybrids synthesized by Yang et al. [100] showed that monosubstituted (either OH or OCH_3_) cinnamoyl-moiety-containing hybrids exhibited inferior antiproliferative activity compared to the disubstituted (with both OH and OCH_3_) cinnamoyl-moiety-containing hybrid compounds, such as compound **8.** These findings demonstrate that increasing the number of electron-donating and electron-withdrawing groups in the hybrids (especially methoxy groups) can enhance their cytotoxic effects, as these groups improve the interaction of the drugs with cancer cell lines.

### 5.2. Impact of Bulky Groups on the Anticancer Activity of FA and CA Hybrids

The in vitro findings emphasized that the introduction of bulky groups, such as aromatic rings, can improve the antiproliferative activity of the hybrid compounds. Thus, in the hybrid compounds synthesized by Malik et al. [111], an extra aromatic ring in hybrids **13a**–**d** was responsible for their superior antiproliferative activity, as hybrid **13d**, with two aromatic rings, exerted an inferior cytotoxic effect compared to compounds **13a**–**c**, with three aromatic rings. Moreover, consistent observations were reported by Sehrawat et al. [116], where SAR evaluations revealed that the introduction of bulky groups (aromatic rings) in hybrid **15** resulted in enhanced antiproliferative activity. Additionally, another study by Sehrawat et al. [117] revealed that compounds with aromatic esters exhibited greater anticancer activity compared to those with aliphatic esters. Moreover, Sucu et al. [119] also revealed that the replacement of the phenyl ring with a 2-furyl ring improved the anticancer activity of the compounds. Hence, the introduction of bulky rings into hybrids can be a potential approach resulting in improved anticancer agents.

### 5.3. Influence of the Linkers on the Anticancer Activity of FA and CA Hybrids

The linkers were reported to influence the anticancer effect of the reported FA and CA hybrids in this review. For instance, the presence of the amide linker in hybrids **7a**–**b** improved the binding energies of the compounds with Ser228 [99]. Additionally, replacement of an ester linker by an oxadiazole linker resulted in the improved anticancer activity of hybrid **15**, as this compound displayed 25% stability compared to that of CAPE [113]. However, although the oxadiazole linker improved the stability of the hybrids, substitution of an ester linker with an oxadiazole compromised their anticancer activity [113]. This demonstrates the significance of ester linkers in the anticancer activity of these hybrids. Furthermore, a study by Sehrawat et al. [116] demonstrated that the introduction of the triazole moiety via the carboxylic group of CA led to improved anticancer activity. Additionally, findings from Sucu et al. [119] illustrated that 1,3,4-oxadiazole-linked hybrids displayed better anticancer effects than other 1,2,4-oxadiazoles. These findings emphasize that the type of azole linker used to form the hybrid can influence its anticancer activity.

### 5.4. Influence of the Position of Substituents on the Anticancer Activity of FA and CA Hybrids

A study by Sucu et al. [118] demonstrated that further modification of the catechol moiety of this compound leads to a compound with poor biological activity. Fonseca et al. [101] reported a similar SAR trend to that observed by Silva et al. [102], showing that the presence of a chlorine substituent at different positions of the hydrazone moiety influenced the anticancer activity of these hybrids, with hybrid **9**, containing chlorine at the para-position, displaying superior activity to its counterparts with a chlorine group at the meta- and ortho positions. This highlights the importance of halogenating the catechol moiety at different positions. Additionally, carboxyl-substituted compounds showed the best anticancer activity compared to their counterparts against HeLa cells. This emphasizes the influence of the position of substitution during hybridization, as compounds substituted at the carboxyl group had superior anti-HeLa activity compared to those substituted at the hydroxyl group of FA [95].

### 5.5. Influence of Alkyl Chain Length on the Anticancer Activity of FA and CA Hybrids

Anticancer activity was also influenced by the alkyl chain length between the two moieties, with compounds synthesized by Wu et al. [95] and Lukáč et al. [96], through a combination of CA and phosphonium salts, illustrating that longer chain lengths exhibited superior anticancer activity compared to those with shorter chains. These studies highlighted that increasing the alkyl chain length between the two combined scaffolds resulted in improved lipophilicity, leading to enhanced anticancer activity.

### 5.6. Influence of Protecting Groups on the Anticancer Activity of FA and CA Hybrids

FA and CA hydroxyl groups are highly reactive and unstable. Hence, protective groups are utilized to overcome these two factors. Cybulsk et al. [99] used different protecting groups in their study, including acetyl (OAc), silyl, and allyl protective groups. The findings showed that the acetyl protective groups were more favorable than silyl and allyl, as these resulted in poor yields, a mixture of products, and degradation of the final products due to amide cleavage as a result of the reagents used for deprotection. Additionally, in another study, Cybulsk et al. [105] used acetyl and allyl protective groups, and the deprotection of the allyl group led to poorer yields compared to those obtained with the acetyl group. Notably, in the study by Lukáč et al. [96], the hydroxyl groups were protected by the acylating agent to allow full completion of the reaction without production of side products and to improve the water solubility of these hybrid compounds. These findings demonstrate the significance of choosing and using a good protecting group when hybridizing compounds with highly reactive and unstable functional groups, with the acetyl protecting group regarded as a priority protecting group. A study by Cybulsk et al. [99] illustrates the importance of deprotection, as the protecting group can influence the anticancer activity of the hybrids. For instance, the acetyl protecting group’s presence on hybrid **7a** compromised its anticancer activity compared to free hydroxyl groups on hybrid **7b**. This emphasizes the importance of the hydroxyl group in these compounds, as this functional group is responsible for the hydrophilicity of these compounds [81,99]. Additionally, it was also highlighted that the direct synthesis of the acetyl-protected precursors leads to acetamide impurities. Hence, converting the acids to acid chlorides is an alternative approach, since chlorine is highly reactive and a good leaving group.

The SAR analysis highlights that the potential anticancer activity of FA and CA hybrids is strongly influenced by multiple structural factors (summarized in Table 13). Functional groups such as methoxy, hydroxyl, and halogens enhance activity by improving molecular interactions with biological targets, particularly when present in combination. The introduction of bulky aromatic groups generally increases potency, likely due to improved binding interactions, while the nature and type of linkers (e.g., amide, ester, oxadiazole, triazole) significantly affect both activity and stability. Additionally, the position of the substituent plays a critical role, with certain positions (e.g., para-substitution) yielding superior activity. Alkyl chain length also contributes, where longer chains enhance lipophilicity and anticancer effects. Furthermore, protecting groups are important for stability and synthesis, although they can influence biological activity, depending on whether key functional groups remain accessible. Overall, these findings demonstrate that anticancer activity is governed by a complex interplay of structural features rather than a single modification.

## 6. Conclusions and Future Perspectives

FA- and CA-based hybrid compounds have emerged as promising scaffolds for the development of compounds with potential anticancer properties, largely due to their ability to integrate multiple pharmacophores into a single molecular framework. This hybridization strategy has demonstrated potential in addressing key limitations of conventional chemotherapy, including poor selectivity, drug resistance, and suboptimal pharmacokinetic properties. Across the studies reviewed, several FA and CA hybrids exhibited moderate to strong in vitro antiproliferative and cytotoxic effects, in some cases comparable to or exceeding those of reference drugs, while also showing improved selectivity toward cancer cells and acceptable drug-likeness profiles.

Mechanistically, these hybrids act through diverse and often complementary pathways, including apoptosis induction, modulation of oncogenic signaling pathways (e.g., PI3K/Akt, EGFR, and mTOR), mitochondrial dysfunction, and enzyme inhibition. Such multitarget activity reinforces the rationale for hybrid design in tackling complex diseases like cancer. Additionally, structural modifications involving linkers (cleavable vs. non-cleavable), substituents (e.g., methoxy and halogens), and functional groups (e.g., hydroxyl and catechol moieties) have been shown to influence biological activity, stability, and pharmacokinetic behavior.

However, despite these encouraging findings, several limitations remain. A significant proportion of the reported studies rely heavily on in vitro assays and in silico predictions, with limited in vivo validation and insufficient exploration of pharmacokinetics and long-term toxicity. Concerns such as potential cardiotoxicity (e.g., hERG inhibition) and inconsistent selectivity profiles highlight the need for more comprehensive safety evaluations.

Furthermore, in some cases, SAR interpretations lack sufficient experimental support, limiting the robustness and generalizability of the proposed design principles.

To advance this field, future research should prioritize the generation of systematic and comparative experimental datasets to robustly validate SAR trends. Greater emphasis should be placed on investigating the interplay between multiple structural modifications, rather than evaluating single-factor effects in isolation. In addition, expanding in vivo studies is essential, particularly those addressing pharmacokinetics and comprehensive toxicological profiling to assess clinical relevance. A deeper exploration of mechanistic pathways is also required to establish clear structure–mechanism relationships. Ultimately, the development of rational design strategies guided by integrated SAR and biological data will be crucial for translating these compounds into viable therapeutic candidates.

In conclusion, while FA and CA hybrid compounds represent a compelling and versatile platform for the development of potential anticancer therapeutics, their successful translation into clinically viable therapeutics will depend on more rigorous experimental validation, deeper mechanistic understanding, and comprehensive evaluation of safety and efficacy.

## Figures and Tables

**Table 1:** In vitro cytotoxic activity (IC_50_, µM) of FA–curcumin hybrid compound (**3**) compared with FA, curcumin, and cisplatin against multiple cancer cell lines (SGC7901, MCF-7, HepG2, A549, and HeLa) and normal cells (MCF-12A).

Hybrids	In Vitro IC_50_ Values (µM)					
	SGC7901	MCF7	HepG2	A549	HeLa	MCF-12A
**3**	23.45 ± 1.52	18.37 ± 1.44	8.38 ± 1.03	1.25 ± 0.22	12.34 ± 1.26	>200
FA	54.43 ± 3.24	34.22 ± 3.15	45.15 ± 3.44	9.15 ± 0.65	41.32 ± 2.35	>200
Curcumin	34.60 ± 2.35	29.04 ± 1.34	38.41 ± 2.93	4.56 ± 0.32	29.72 ± 2.42	>200
Cisplatin	1.25 ± 0.41	2.34 ± 0.33	4.62 ± 0.83	3.63 ± 0.76	4.32 ± 0.85	ND

**Table 2:** In vitro cytotoxic activity (IC_50_, µM) of chromene–oxadiazole FA hybrid compounds (**6a**–**c**) compared with doxorubicin against HEK-293, HEPG2, B16, SKBR3, and DU145 cell lines.

Hybrids	In Vitro IC_50_ Values (µM)				
	HEK-293	HEPG2	B16	SKBR3	DU145
**6a**	44.921 ± 0.5	26.237 ± 3.9	76.133 ± 1.7	90.556 ± 2.0	54.507 ± 5.1
**6b**	15.311 ± 2.1	26.008 ± 2.4	24.156 ± 0.6	34.020 ± 3.7	28.420 ± 1.5
**6c**	29.376 ± 0.8	34.829 ± 1.2	42.051 ± 1.1	70.260 ± 2.6	43.924 ± 3.1
Doxorubicin	6.12 ± 0.5	0.72 ± 0.012	0.45 ± 0.52	0.7 ± 0.56	2.5 ± 1.42

**Table 3:** In vitro cytotoxic activity (IC_50_, µM) and selectivity index (SI) of CA-based hybrid compounds (**7a**–**b**) compared with capecitabine and 5-fluorouracil against pancreatic cancer cell lines (AsPC-1 and BxPC-3) and normal human dermal fibroblasts (NHDFs).

Hybrids	In Vitro IC_50_ Values (µM)			SI	
	AsPC-1	BxPC-3	NHDFs	AsPC-1	BxPC-3
**7a**	57.15 ± 2.06	13.60 ± 2.48	96.49 ± 5.79	1.69	7.10
**7b**	132.60 ± 5.67	23.16 ± 1.25	163.40 ± 4.33	1.23	7.06
Capecitabine	>200	>200	>200	–	–
5-Fluorouracil	52.39 ± 11.56	12.74 ± 2.73	>200	–	–

**Table 4:** In vitro cytotoxic activity (IC_50_, µM) of FA-based hybrid compound (**8**) compared with chloropyramine against triple-negative breast cancer cell lines (MDA-MB-231, MDA-MB-157, and MDA-MB-453) and normal breast epithelial cells (MCF-10A).

Hybrids	In Vitro IC_50_ Values (µM)			
	MDA-MB-231	MDA-MB-157	MDA-MB-453	MCF10A
**8**	8.37	12.09	9.07	40.63
Chloropyramine	49.73	43.06	>60	>60

**Table 5:** In vitro cytotoxic activity (IC_50_, µM) of FA-based hybrid compound (**9**) compared with cisplatin against lung cancer cell lines (A549 and H1299).

Hybrids	In Vitro IC_50_ Values (µM)	
	A549	H1299
**9**	23.68 ± 0.66	32.15 ± 0.96
Cisplatin	28.13 ± 1.65	34.51 ± 1.20

**Table 6:** In vitro cytotoxic activity (IC_50_, µM) of CA–metformin hybrid compound (**10**) compared with cisplatin against breast (MDA-MB-468) and lung (A549) cancer cell lines.

Hybrids	In Vitro IC_50_ Values (µM)	
	MDA-MB-468	A549
**10**	5.47 ± 2.72	4.42 ± 2.15
5-Fluorouracil	2.73 ± 0.56	84.04 ± 2.61

**Table 7:** In vitro antiproliferative activity (IC_50_, µM) of FA–tetrahydroisoquinoline hybrid compound (**11**) compared with gefitinib against cancer cell lines (A549, HepG2, HCT-116, and HL-60) and normal endothelial cells (HUVECs).

Hybrids	In Vitro IC_50_ Values (µM)				
	A549	HepG2	HCT-116	HL-60	HUVECs
**11**	2.45 ± 0.16	1.01 ± 0.11	5.79 ± 0.41	2.18 ± 0.14	>50
Gefitinib	1.86 ± 0.13	0.96 ± 0.07	5.33 ± 0.51	2.42 ± 0.20	>50

**Table 8:** In vitro cytotoxic activity (IC_50_, µM) of CA-based hybrid compound (**12**) against AsPC-1, BxPC-3, HeLa, and MCF-7 cancer cell lines.

Hybrids	In Vitro IC_50_ Values (µM)			
	AsPC-1	BxPC-3	HeLa	MCF-7
**12**	336.50 ± 85.01	347.53 ± 52.39	203.15 ± 35.28	748.45 ± 12.23
DiMIQ	1622.50 ± 131.98	888.77 ± 49.52	784.20 ± 20.03	868.30 ± 18.81

**Table 9:** In vitro cytotoxic activity (IC_50_, µM) of FA–triazole hybrid compounds (**13a**–**d**) compared with reference drugs (tamoxifen and erlotinib) against breast (MCF-7) and lung (A549) cancer cell lines.

Hybrids	In Vitro IC_50_ Values (µM)	
	MCF-7	A549
**13a**	118.24	478.32
**13b**	89.17	561.12
**13c**	108.69	712.85
**13d**	562.13	867.92
Tamoxifen	392.3	Not tested
Erlotinib	Not tested	582.73

**Table 10:** In vitro cytotoxic activity (IC_50_, µM) of CA-based hybrid compounds **15** and **16** compared with methotrexate and doxorubicin against breast cancer cell lines (MCF-7 cells).

Hybrids	In Vitro IC_50_ Values (µM)
	MCF-7 Cell
**15**	8.53 ± 0.19
**16**	5.37 ± 0.16
Methotrexate	18.96 ± 2.13
Doxorubicin	3. 62 ± 0.02

**Table 11:** In vitro cytotoxic activity (IC_50_, µM) of CA-derived hybrid compounds (**17a**–**b**) compared with CA phenethyl ester (CAPE) against glioblastoma cell lines (T98G and LN229).

Hybrids	In Vitro IC_50_ Values (µM)	
	T98G	LN229
**17a**	48.19	21.94
**17b**	46.42	14.23
CAPE	97.92	112.5

**Table 12:** In vitro cytotoxic activity (IC_50_, µM) of hybrid compounds (**18a**–**b**) compared with CA and CA–phenethyl ester (CAPE) against multiple cancer cell lines, including A549, U87, SKOV3, LN229, MCF-7, and T98G.

Hybrids	In Vitro IC_50_ Values (µM)					
	A549	U87	SKOV3	LN229	MCF-7	T98G
**18a**	62	60.3	21.1	80.4	70.9	39.2
**18b**	18.3	35.1	14.2	37.9	30.9	34.4
CAPE	191.3	97.1	35.5	118.2	61.2	97.9
CA	74.9	Not detected	38.4	56.6	46.1	51.5

**Table 13:** Summary of SAR findings for FA- and CA-based hybrid compounds reported between 2022 and 2025, highlighting modification sites, linker types, and key structural features influencing anticancer activity.

Hybrids	Modification Sites	Linker Types	SAR Findings	Ref.
**2**	Carboxylic group	Esters	Introducing more methoxy groups on the side chain of the aromatic ring and replacing the aromatic ring with indole enhanced the anticancer activity.	[92]
**3**	Carboxylic group	Esters	No obvious trend	[94]
**4**	Carboxylic and hydroxyl	Esters	Monosubstituted hybrids were more biologically active than disubstituted hybrids, illustrating that the disruption of the hydroxyl group compromised the anticancer activity. Moreover, longer chain length was more favorable than short chains between the moieties.	[95]
**5**	Carboxylic and hydroxyl groups	Esters	Prolongation of the alkyl chain length between two moieties enhanced the anticancer activity.	[96]
**6**	Carboxylic group	Amides and azoles	The presence of methoxy or bromo groups on either side of the hybridized moieties positively influenced the anticancer activity.	[97]
**7**	Carboxylic and hydroxyl groups	Amides	Amide linkers and free hydroxyl groups enhanced the anticancer activity	[99]
**8**	Carboxylic group	Amides	Disruption of the hydroxyl group compromised the anticancer activity.	[100]
**9**	Carboxylic group	Amides	The position of chlorine influenced the anticancer activity	[101]
**10**	Carboxylic acid group	Amides	No obvious trend.	[103]
**11**	Carboxylic and hydroxyl groups	Amides and esters	No obvious trend.	[104]
**12**	Carboxylic group	Amides	No obvious trend.	[105]
**13**	Carboxylic group	Azoles	The presence of an extra aromatic ring improved the anticancer activity.	[111]
**14**	Carboxylic group	Azoles	Replacing the ester linker with an oxadiazole linker compromised the anticancer activity.	[113]
**15**	Carboxylic group	Azoles	Introduction of bulky aromatic rings enhanced the anticancer activity of the hybrids.	[116]
**16**	Carboxylic group	Azoles	Introduction of bulky aromatic rings and ester linkers enhanced the anticancer activity of the hybrids.	[117]
**17**	Carboxylic group	Azoles	Azole linkers were more favorable than amide linkers.	[118]
**18**	Carboxylic group	Oxazole	Replacing OCH_3_ with OCF_3_ compromised the anticancer activity, and 2-furyl-containing compounds were more active than phenyl-containing hybrids.	[119]

## Data Availability

Not applicable.

## Abbreviations

The following abbreviations are used in this manuscript:

FA	Ferulic Acid
CA	Caffeic Acid
SAR	Structure–Activity Relationship
ADMET	Absorption, Distribution, Metabolism, Excretion, and Toxicity
BBB	Brain–Blood Barrier
CNS	Central Nervous System
FDC	Fixed-Dose Combination
GI	Gastrointestinal
TPSA	Topological Polar Surface Area
P-gp	P-glycoprotein
IC50	Half Maximal Inhibitory Concentration
TNBC	Triple-Negative Breast Cancer
CAPE	Caffeic Acid Phenethyl Ester
EGFR	Epidermal Growth Factor Receptor
ER-a	Estrogen Receptor alpha
